# Neurohumoral Profiles and Childhood Adversity of Patients with Multisomatoform Disorder and Pain as the Leading Bodily Symptom

**DOI:** 10.1155/2022/7958375

**Published:** 2022-02-22

**Authors:** Johannes Achenbach, Lilly Volkmann, Anh-Thu Tran, Burkard Jäger, Mathias Rhein, Alexander Glahn, Michael Bernateck, Matthias Karst

**Affiliations:** ^1^Department of Anesthesiology and Intensive Care Medicine, Pain Clinic, Hannover Medical School, Carl-Neuberg-Str. 1, 30625 Hannover, Germany; ^2^Klinikum Region Hannover, Nordstadt Krankenhaus, Department of Anaesthesiology, Intensive Care Medicine, Intensive Care Medicine, Pain Medicine, Haltenhoff str. 41, 30167 Hannover, Germany; ^3^Klinik für Kinderchirurgie und Kinderneurologie, Klinikum Bremen Mitte, St.-Jürgen-Str. 1, 28177 Bremen, Germany; ^4^Department of Neurology and Neurophysiology, Hannover Medical School, Carl-Neuberg-Str. 1, 30625 Hannover, Germany; ^5^Department of Psychosomatics and Psychotherapy, Hannover Medical School, Carl-Neuberg-Str. 1, 30625 Hannover, Germany; ^6^Laboratory for Molecular Neuroscience, Department of Psychiatry, Social Psychiatry and Psychotherapy, Hannover Medical School, Carl-Neuberg-Str. 1, 30625 Hannover, Germany; ^7^Center of Pain Medicine Hannover, Aegidientorplatz, 30159 Hannover, Germany

## Abstract

**Objective:**

Patients suffering from chronic pain often present with multifactorial underlying conditions, sometimes without concrete pathological physical findings. Functional somatic syndromes (FSS) and somatoform disorders show a high prevalence of 8-20% and are often associated with adverse childhood experiences (ACE) and chronic stress. As many different FSS have overlapping symptoms, the concept of multisomatoform disorder (MSD) has been introduced as an encompassing concept. We hypothesize that a common neurohumoral profile is present in patients with MSD that is distinct from gender- and age-matched controls and thus provides insight into possible common underlying mechanisms.

**Design:**

In 151 patients with MSD (138 females) and 149 matched controls (131 females), we determined ACE by the Childhood Trauma Questionnaire (CTQ) and chronic stress by the Trier Inventory for Chronic Stress (TICS). Furthermore, the serum levels of leptin, FSH, LH, cortisol, DHEA-S, and IGF-1 have been assessed.

**Results:**

There were significant differences in the levels of leptin, FSH, IGF-1, and cortisol between patients and controls, mainly driven by female participants. Levels of leptin were significantly correlated with BMI in patients, in controls, and in the female subgroup. This correlation was exaggerated in female patients when compared to female controls. Both CTQ and TICS predicted MSD directly and indirectly through the levels of leptin.

**Conclusion:**

There is evidence of a distinct neurohumoral profile in female patients with MSD when compared to matched healthy controls, similar to what has been demonstrated in other chronic pain states. The observed profile can be taken as possible evidence for a dysregulated response to chronic stress and metabolic balance as well as a state of hypocortisolism and HPA-axis dysfunction. ACE and chronic stress play a major role in the development of MSD and altered neurohumoral profile.

## 1. Introduction

Causes of chronic pain are often debilitating and multifactorial in origin while often lacking physical findings which adequately explain the presenting symptoms. Somatoform disorders, a DSM-IV classification, and functional somatic symptoms (FSS) are among the most challenging of the possible etiologies with a prevalence of 8-20% [1–3]. In FSS such as fibromyalgia syndrome (FMS) or irritable bowel syndrome (IBS), physical findings that explain the presenting symptoms are absent even after comprehensive examinations and diagnostics. Distressing functional and disabling physical symptoms are often combined with severe chronic pain as the most prominent clinical complaint. This constellation of symptoms is also present in multisomatoform disorder (MSD), which was originally suggested by Kroenke et al. and serves as a common point of reference for patients in different somatic and psychosomatic specialties [1, 3]. Despite not being included in the ICD-10 or DSM-V, the concept of MSD is useful in studying large well-characterized cohorts of prototype functional somatic symptoms and was used to recruit patients with severe functional disability from chronic pain [3]. MSD is defined as three or more current disabling functional somatoform symptoms (i.e., pain, dizziness, and bowel dysfunction) unexplained by organic disease or other mental disorders. Symptoms should be present at least half of the days over a two-year period and be associated with increased healthcare utilization [1, 3]. Further criteria are the exclusion of severe psychiatric disorders (i.e., substance abuse, schizophrenia) and significant cognitive impairment [2]. There is a high prevalence of pain and pain-associated disability in patients with MSD as evidenced by pathologically altered scores in health questionnaires and elevated VAS scores compared with controls and other FSS or psychiatric disorders [1, 4].

Presently, the pathophysiology of chronic pain in somatoform disorders and FSS is incompletely understood. Negative influences on allostatic load through environmental, developmental, and genetic factors as well as physiological and psychological stressors have been suggested [5]. As part of the effort to understand chronic pain in somatic syndromes, single nucleotide polymorphisms (SNPs) of genes have been studied with inconsistent results. Previous studies suggest a role of SNPs in serotonergic and dopaminergic but not catecholamine-o-transferase genes in the etiology of MSD [6–8]. Patients with MSD and pain as the leading bodily symptom show characteristic sensory alterations as demonstrated through quantitative sensory measurements [9]. In human physiology, a complex interplay between an acute and chronic response to stress, hormonal regulation of homoeostasis, and neurotrophic factors exists. In human and animal studies, it has been shown that adverse childhood experiences (ACE) play a major role in the development of FSS and somatoform disorders [10, 11] and are associated with susceptibility to painful conditions [12, 13].

Higher degrees of childhood traumatization have been associated with increased salivary cortisol levels after a laboratory stressor, a possible sign of a heightened stress response [14]. In contrast, chronic stress has also been found to impair cortisol secretion and HPA-axis (hypothalamic-pituitary-adrenal axis) function in general as well as in patients with fibromyalgia leading to decreased resilience [15, 16]. Expanding on the notion of endocrine alterations, male and female levels of cortisol, LH (luteinizing hormone), FSH (follicle stimulating hormone), DHEA-S (dehydroepiandrosterone sulfate), and IGF-1 (insulin-like growth factor) are influenced by the HPA axis. They are also associated with obesity and chronic painful conditions [17, 18].

The proteohormone leptin is involved in body homoeostasis, energy expenditure, and appetite [19]. It plays a further role in the context of stress physiology through interaction with hypothalamic-pituitary as well as sympathoadrenergic levels [20, 21]. We hypothesized that levels of neuroendocrine and neurotrophic profiles are significantly altered in patients with MSD representing chronic maladaptation of stress response and homeostasis as proposed for FMS [15]. In a matched case-control study, we examined the neuroendocrine profiles of 149 controls and 151 patients with MSD and pain as the leading symptom compared with healthy volunteers.

## 2. Materials and Methods

### 2.1. Subjects

The patient collective was previously investigated regarding the role of single nucleotide polymorphisms [6–8] as well as the difference in quantitative sensory profiles and epigenetic regulation of TRPA1 expression between patients and controls [9, 11]. Altogether, 300 individuals were recruited to participate (151 patients, 149 controls). Participants were recruited at the outpatient pain clinic of the Hannover Medical School, Hannover, Germany; at the Clinic for Psychosomatic Medicine and Psychotherapy of the Hannover Medical School; and at several fibromyalgia support groups. Healthy age- and gender-matched controls not experiencing pain at time of recruitment were selected at the same time. A basic assessment through psychometric questionnaires (SF-36, PHQ-15, German 28-item version of the Childhood Trauma Questionnaire, and Post-Traumatic Stress Diagnostic Scale) as well as a complete clinical examination took place at the time of recruitment after severe psychiatric or somatic conditions were ruled out by clinician experts. The chief presenting complaint of all patients was chronic widespread pain with its intensity reported using the visual analogue scale (VAS). Patients were screened for possible MSD through demonstrating a PHQ-15 score > 10 and a 36-Item Short-Form 36 (SF-36) physical component score ≤ 40 which demonstrates strong psychophysiological strain as established previously [3]. Additionally, a modified interview of the somatoform disorder section of the Structured Clinical Interview of the DSM-IV (SCID) was used [1, 3, 6–8]. The interview (SCID) was modified to check for published diagnostic criteria of MSD [1, 3] and required three currently present somatoform symptoms in addition to one pain-related symptom which are functionally disabling. They need to present a significant impairment of the physical quality of life and to be insufficiently explained by organic diseases or mental disorders. Somatoform symptoms needed to be present in half of the days over the previous two years and resulted in the utilization of healthcare resources.

Additionally, participants answered all 28 items of the Childhood Trauma Questionnaire (CTQ) on a five-point rating scale (1 = “not at all” to 5 = “very much”). The CTQ subscales describe emotional abuse, physical abuse, sexual abuse, emotional neglect, and physical neglect. Subscale scores are computed by summing up the score of the individual items. This results in a score with a range between 5 and 25 points. The resulting score is then categorically rated from no trauma to extreme trauma [22]. To differentiate between participants with severe multiple trauma events and mild or no trauma, we first binned the resulting subscale categories: none to mild trauma (≤2) and severe trauma (>2) resulting in two scores (0 or 1). We then added these scores (possible summary result range: 0 to 5) and split the participants in three groups: no (0 points), mild (1–2 point), and severe (>2 points) trauma as previously described [11, 23].

Furthermore, participants answered the Trier Inventory for Chronic Stress (TICS). This is a standardized questionnaire with 57 items for the differential diagnosis of 9 different facets of chronic stress (work overload, social overload, pressure to succeed, job dissatisfaction, excessive demands at work, lack of social recognition, social tension, social isolation, and chronic anxiety). There is no determined cutoff for critical stress in the TICS questionnaire. A high score correlates with a higher exposure to stress, whereas a low score shows infrequent exposure to stress.

Exclusion criteria were defined as age < 18 years, insufficient German language ability, insufficient cognitive abilities, severe and chronic somatic diseases (e.g., severe heart failure, encephalitis disseminate, and dementia), and severe comorbid mental disorders which cause major impairment of social functioning (e.g., schizophrenia, severe mood disorders, personality disorders, and substance abuse). The revised Declaration of Helsinki in 2000 (Edinburgh, 52nd general meeting) was followed in all investigations. After approval by the Ethical Committee of Hannover Medical School (study protocol number 4757), informed consent was obtained from all subjects for blood sampling, genotyping, and clinical measurements [6–8].

### 2.2. Serum Parameters

Blood was collected between 8:00 and 9:00 am for each participant to be in keeping with the circadian rhythm of hormone release of the HPA axis. EDTA vials (4 ml) and serum vials (5 ml) were used (S-Monovette, Sarstedt). Measurements were performed through the Department of Endocrinology of the Hannover Medical School (MHH).

### 2.3. Cortisol

A competitive immunoassay (direct chemiluminescence technique, Siemens, Germany) using polyclonal rabbit anti-cortisol antibodies and mouse-anti-rabbit antibodies was used. Quantification was performed via the ADVIA Centaur system.

### 2.4. LH (Luteinizing Hormone)

A sandwich immunoassay with direct chemiluminescence (Siemens, Germany) using paramagnetic particle-bound monoclonal mouse-anti-LH antibodies marked with acridinium ester was used. Quantification was performed via the ADVIA Centaur system.

### 2.5. FSH (Follicle Stimulating Hormone)

A sandwich immunoassay with direct chemiluminescence (Siemens, Germany) using polyclonal sheep-anti-FSH antibodies and monoclonal mouse-anti-FSH antibodies bound to paramagnetic particles was used. Quantification was performed via the ADVIA Centaur system.

### 2.6. IGF-1 (Insulin-Like Growth Factor-1)

An enzyme-marked solid-phase immunometric chemiluminescence assay (Siemens, Germany) was used. Monoclonal mouse-anti-IGF-1 antibodies and polyclonal rabbit-anti-IGF-1 antibodies were utilized. Quantification was performed via the Immulite 2000 System (Siemens, Germany).

### 2.7. DHEA-S (Dehydroepiandrosterone Sulfate)

A competitive chemiluminescence assay (Fa. DiaSorin, Saluggia, Italy) was utilized using monoclonal mouse-anti-DHEA-S antibodies. Quantification was performed using the LIAISON analyzer (Fa. DiaSorin, Saluggia, Italy).

### 2.8. Leptin

A radioimmunoassay was performed using the human leptin RIA kit (LINCO research, St. Charles, Missouri, USA).

### 2.9. Statistical Analysis

Analysis was performed using SPSS Statistics (IBM Corp. Released 2017, IBM SPSS Statistics for Macintosh, Version 25.0. Armonk, NY: IBM Corp). Differences between patients and controls were assessed using a two-sided *t*-test for independent samples. Adjustment for multiple comparisons was not made as comparisons were preplanned before the study was commenced and the selection of serum parameters was based on biological reasoning as well as existing literature. This is in contrast to exploratory genome-wide association studies where adjustment for multiple comparisons is required. Equality of variance was determined using the Levene test. Interpretation of the results was conducted accordingly. Results are given as mean and standard deviation. Due to well-known sex-specific alterations in hormone levels, the analysis was performed according to gender as opposed to the patient and control groups as a whole. Moreover, considering the much larger number of female participants, most of the signal will be generated by female participants. Hormone levels were also analyzed using one-way ANOVA according to medication use. Additionally, the influence of BMI on leptin levels between patients and control subjects was performed using Spearman correlation analysis. Spearman correlation was also used to further characterize the association of stress and childhood trauma with hormone levels. Furthermore, mediation analysis was used to examine if levels of cortisol and leptin had mediating effects on the development of “patientness” caused through chronic stress and childhood trauma as measured through the CTQ and TICS questionnaires. Calculations were performed using the Process 3.5.3 macro for SPSS by A. Hayes.

## 3. Results

Three hundred participants were recruited for the study (151 patients, 149 controls). Group characteristics are shown in Table 1. Two participants (1 control, 1 patient) did not complete the entire study so that hormone levels of cortisol, LH, FSH, IGF-1, and DHEA-S were analyzed in 148 controls and 150 patients. Leptin levels were measured in only 244 participants (141 controls, 103 patients). Patients and controls did not differ significantly with regard to demographic characteristics; SF-36 scores differed significantly between patients and controls, as expected, as it was part of the inclusion criteria in patient selection.

**Figure 1:**
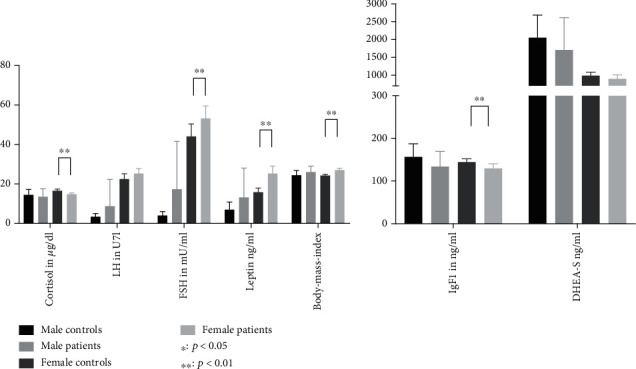
Mean hormone levels and BMI of patients and controls separated according to gender. Graphs represent mean ± 95%confidence interval.

Hormone levels were analyzed between male and female patients and controls. Significant differences between patients and controls were observed only for female participants. However, despite not reaching statistical significance, the directional trend of alterations in male participants appears to be similar to female participants.  Table 2 shows the mean values and standard deviations for the measured hormone levels as well as BMI. A graphical representation is shown in Figure 1.

No differences between patients and controls were found in male participants with regard to BMI or any hormone level investigated. Due to the small sample size of male participants providing insufficient explanatory power and in the absence of statistically significant findings, further investigation focused mainly on female study participants.

### 3.1. Cortisol

Significantly lower cortisol levels were found in female patients compared to controls (control vs. patients: mean, SD (*μ*g/dl); 16.55 ± 4.91 vs. 14.76 ± 4.55, *t*(265) = 3.101, *p* = 0.002).

### 3.2. LH

No significant differences were found between female patients and controls (control vs. patients: mean, SD (U/l); 22.40 ± 15.85 vs. 25.20 ± 15.26, *t*(265) = −1.471, *p* = 0.142).

### 3.3. FSH

Significantly higher levels were found in female patients with regard to FSH (control vs. patients: mean, SD (mU/ml); 44.08 ± 36.67 vs. 53.16 ± 37.38, *t*(265) = −2.001, *p* = 0.046).

### 3.4. IGF-1

Significantly lower levels were found in female patients (control vs. patients: mean, SD (ng/ml); 144.61 ± 44.18 vs. 129.64 ± 64.81, *t*(265) = 2.194, *p* = 0.029).

### 3.5. DHEA-S

There were no significant differences found between female patients and controls (*t* [24] = 1.220, *p* = 0.224).

### 3.6. Leptin and BMI

We found significantly higher serum leptin levels in female patients compared to controls (control vs. patients: mean, SD (ng/ml); 15.76 ± 12.13 vs. 25.24 ± 18.08, *t*(218) = −4.654, *p* = <0.001).

A statistically significant difference between the mean BMI of female patients and controls was found (control vs. patients: mean, SD (kg/m^2^); 24.20 ± 3.96 vs. 27.02 ± 5.26, *t*(246) = −4.766, *p* = <0.001).

**Figure 2:**
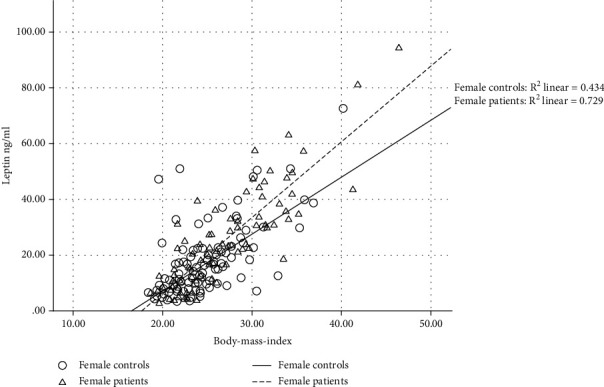
BMI is plotted against serum leptin levels in female patients and controls. A strong correlation was found in female controls (*R*^2^ = 0.434) and even stronger in female patients (*R*^2^ = 0.729) with a significance level of *p* < 0.0001 in Spearman correlation analysis.

As patients and control groups differed significantly with regard to BMI and due to the significant influence of weight on hormone levels in general and leptin levels in particular, we analyzed the BMI distribution in female patients and controls. Despite statistical significance, there is only a marginal shift to the right observable in the cumulative histogram plot (supplementary fig. S1) with questionable clinical significance. On further investigation, we investigated the correlation of leptin levels and BMI. Here, a strong correlation was found between BMI and leptin in female controls (*R*^2^ = 0.434) and even stronger in female patients (*R*^2^ = 0.729) (Figure 2) with a significance level of *p* < 0.0001 in Spearman correlation analysis.

### 3.7. Medication Use

Data on medication use was missing in 19 (7 in controls, 12 in patients) of 269 female participants. Table S1 shows the usage of conventional analgesics, opioids, anticonvulsants, and antidepressants in patients and controls. Further examination revealed that only 23 female patients vs. 111 controls took no medication. Conventional analgesics only were used by 36 female patients and 11 female controls. Centrally acting medications (any or a combination of opioids, antidepressants, and anticonvulsants) were used by 67 female patients and only two female controls. In female patients, these three groups (no medication, conventional analgesics only, and centrally acting medication) did not differ regarding measured hormone levels or BMI using a one-way ANOVA (data not shown).

### 3.8. Influence of Stress and Childhood Traumatization on Hormone Levels

In female participants, the cumulative TICS score was significantly correlated positively with leptin levels (*R*^2^ = 0.264, *p* < 0.01) and negatively with cortisol levels (*R*^2^ = −0.194, *p* < 0.01). No such correlation existed for CTQ sum and subscores.

**Figure 3:**
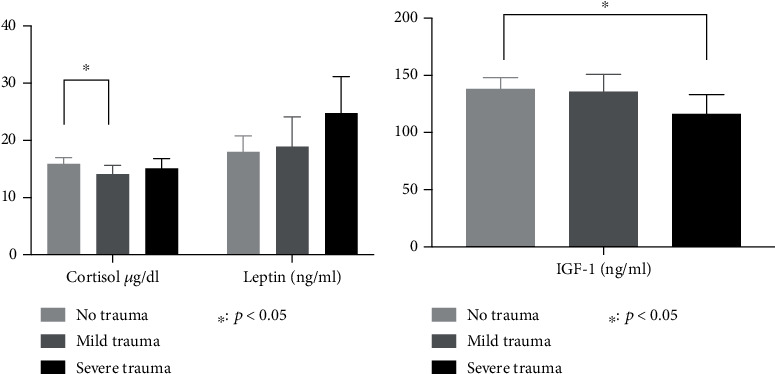
Graphical representation of the one-way ANOVA between-group comparison (trauma levels no, mild, and severe) of cortisol (*μ*g/ml), leptin (ng/ml), and IGF-1 (ng/ml). Differences between no and severe trauma were significant regarding IGF-1 and between no and mild trauma for cortisol. Leptin levels showed a strong trend towards a significant difference between no and severe trauma.

Utilizing one-way ANOVA, there was a significant effect of the degree of childhood traumatization (non, mild, and severe) on cortisol (*F* = (2, 248) = 3.24; *p* = 0.041) and IGF-1 (*F* = (2, 248) = 3.318; *p* = 0.038). For leptin levels, the significance level was just missed (*p* = 0.064). A Tukey post hoc test revealed that cortisol levels were significantly lower in the mild compared to the no trauma group (16.208 ± 4.6595 vs. 14.4 ± 4.86, *p* = 0.032) and that IGF-1 levels were significantly lower in the severe compared to the no trauma group (139.82 ± 48.907 vs. 117.78 ± 51.23, *p* = 0.03). With regard to leptin, the difference between no and severe trauma just missed significance (18.29 ± 14.01 vs. 25.08 ± 18.49, *p* = 0.052) (also see Figure 3)

### 3.9. Mediation Effect of Leptin and Cortisol on the Development of MSD through Chronic Stress and Trauma

**Figure 4:**
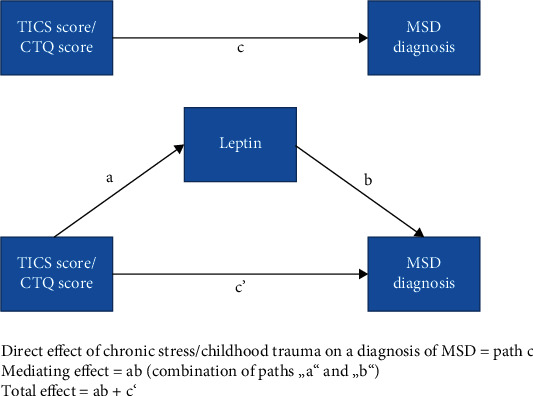
Mediation effect of leptin.

A simple mediation analysis was performed using the PROCESS macro for SPSS V. 3.5.3. to analyze if chronic stress as measured via the TICS questionnaire can predict “patientness” in our sample and if that direct path would be mediated by leptin and or cortisol. A direct effect of the TICS score on the development of MSD was observed, *B* = 0.064, *p* < 0.001 (Figure 4). When entering mediators into the model, TICS score predicted leptin levels as a mediator significantly *B* = 0.1506, *p* = 0.0006, which in turn predicted belonging to the group of patients with MSD, *B* = 0.0386, *p* = 0.0054 (Figure 4). We found that MSD diagnosis from chronic stress is mediated by cortisol levels, ab = 0.0058, 95% CI (0.0011; 0.0130). Mediation through leptin could also be observed on the effects of trauma levels (no, mild, and severe) as measured by CTQ as described above (Figure 4). The direct effect of trauma grade on MSD was significant with *B* = 1.0797, *p* < 0.0001, as was the effect of leptin as the mediator (*B* = 3.0129, *p* = 0.0491), which in turn significantly influenced the diagnosis of MSD (*B* = 0.0461, *p* < 0.0001). The effect of trauma levels on MSD diagnosis is significantly mediated with an indirect effect of ab = 0.1388 95% CI (0.0032; 0.3170).

In dichotomous outcomes, a total effect model is not calculated and thus cannot be contrasted with the direct effect. It is therefore not possible to distinguish between partial and complete mediation. Cortisol did not show mediation effects on stress measured by TICS or childhood trauma grades.

## 4. Discussion

The role of hormonal alterations and stress has been investigated previously in different pain disorders and FMS. Often, the emphasis was only put on classical hormonal markers of stress or gonadal hormones. To our knowledge, this is the first study to investigate leptin, hormones of the HPA axis, and the hypothalamic–pituitary–gonadal axis (HPG) simultaneously in a large collective of patients with MSD. This is of special importance as people with different somatoform disorders often have overlapping symptoms. The concept of the diagnosis of MSD honors that fact. We hypothesized that patients with MSD share a common hormonal profile distinct between patients and controls, hinting at underlying pathophysiological mechanisms. Here, the concept of allostasis and maladaptive response to chronic stress seems to be of paramount importance [5]. We observed significantly lower cortisol levels in female patients than controls, following known data from other chronic pain states and indicating HPA-axis dysfunction. Previous studies demonstrated that prolonged stress and hyperactivity of the HPA axis could result in states of hypocortisolism, alterations of circadian rhythm and function of the HPA axis in animal models, patients with FMS, chronic fatigue, and widespread pain [15, 25, 26]. Critically important in support of these previous data are our findings of a correlation between chronic stress as measured by the TICS questionnaire and lower cortisol levels along with lower cortisol levels in female participants suffering from childhood trauma. This is in concordance with a study in patients with FMS showing decreased diurnal variation and lower morning levels of salivary cortisol similar to our findings [24]. Studies with patients suffering from somatoform disorders showed generally blunted salivary cortisol profiles during the day as well as altered circadian rhythms [27].

McBeth and colleagues demonstrated that in a group of psychologically at-risk subjects, dysfunction of the HPA axis helped distinguish between patients who will or will not develop chronic widespread pain [28]. However, the current data is not unanimous. Accordingly, a recent meta-analysis of 28 studies showed both hyperactivity and hypoactivity of the HPA axis, depending on timing, type of adversity, and subtype of maltreatment [16]. The observed differences can be explained by methodological differences and heterogeneity of the study populations. Another aspect is the time course of the disease investigated. Heightened HPA-axis activity results in higher cortisol levels [24, 25]. During chronic stress with increased allostatic load [29], this system is considered to be “burnt out” with resulting hypoactivity of the HPA axis and resulting decreased hormonal secretion [24, 25].

In addition to alterations of the HPA axis, changes in sex hormone levels have been investigated; this is partially due to the much higher disease prevalence in women and partially due to an indirect influence of alterations of the HPA axis on sex hormone secretion. As testosterone is produced by women mainly in the adrenal gland and is responsible for the production of DHEA-S, DHEA-S has been considered a good marker of adrenocortical hypofunction [30]. Additionally, DHEA-S production is stimulated through ACTH release, as is cortisol production. Lowered levels in patients with FMS have previously been shown in a sample of 56 patients compared with controls [26]. In our study, lower levels of DHEA-S were found in female patients but failed to reach statistical significance level (*p* = 0.224). Regarding FSH, our study was in agreement with a previous investigation that demonstrated higher morning FSH levels with a decrease in estrogen levels in women with FMS [31]. Others have reported unaltered levels of FSH compared with controls [32]. A large study of men found a higher risk of experiencing chronic widespread pain when belonging to the group with the highest FSH and LH group [33]. As cortisol and DHEA-S levels were both lower in patients than in controls, a lack of central stimulation through, i.e., ACTH, could be present as lowered DHEA-S levels as well as cortisol/DHEA-S ratios have been used to predict secondary HPA-axis dysfunction [34]. Further studies with regard to LH levels in patients with FMS are in keeping with previous studies while Riedel et al. observed unaltered LH base levels [31, 32]. However, there was a blunted response to LH-releasing hormone stimulation.

Our study demonstrated lower IGF-1 levels in patients with MSD, similar to previous studies in patients with FMS. IGF-1 levels have been previously found to be lower in patients with FMS [35]. At the same time, one study showed the potential of human growth hormone replacement to increase IGF-1 levels and reduce pain in patients with FMS and proven reduced IGF-1 levels [36]. In treatment studies of patients with FMS and reduced levels of IGF-1, one study found decreased levels of IGF-1 after resistance exercise, while another observed increased levels of IGF-1 after aerobic exercise, both with improvement in clinical symptoms [18]. The complex interplay and sometimes conflicting results point to a significant role of higher controls, especially CRH, as increased CRH levels are associated with FMS [37]. CRH itself stimulates somatostatin release in the hypothalamus, which in turn inhibits GH release [37], and could thus be one possible explanation for gonadal alterations as well as lowered IGF-1 levels in FMS patients.

Lastly, in our study group, leptin levels and BMI were shown to be higher in female patients with MSD. Leptin levels found in blood are generally proportional to body fat and adipocytes in which the hormone is predominantly produced [38]. Leptin resistance has been demonstrated in obesity states [39] as a reactive increase due to leptin receptor downregulation. Leptin also influences the HPA-axis, i.e., lowering cortisol levels after injection in a rat model [20]. A possible mechanism of this is a leptin-mediated decrease in norepinephrine release in the brainstem and paraventricular nucleus, which in turn leads to a decrease in stimulation of corticotropin-releasing hormone release [40]. This is especially plausible, as leptin receptors have been demonstrated in noradrenergic nuclei of the brainstem and the paraventricular nucleus [41]. The clinically observed reduction in cortisol levels could thus be possibly explained [20]. Leptin has also been implied in somatic [42] and functional [43] pain states. It has also been shown to be elevated in patients with FMS independent of body weight [44]. This is especially noteworthy as increased weight and obesity have a high prevalence in patients with FMS [17, 45] which is paralleled by our findings. Among the explanations for a higher prevalence of obesity in patients with MSD are higher rates of comorbid depression [4] which in itself has a higher incidence of above-average body weight. Additionally, chronic pain states often result in a state of fear avoidance and kinesiophobia, resulting in reduced physical activity and consecutive weight gain [46]. Lastly, a medication commonly prescribed in chronic pain states like antidepressants and gabapentinoids often cause weight gain as a common side effect. One could argue that higher leptin levels in female patients of our study population are merely the result of the comparatively higher BMI levels. However, the observed higher correlation of BMI with leptin in patients compared to controls suggests a dysregulated or overactive leptin production caused by increased fat mass, thus favoring an altered stress response and pain perception. The significant correlation of TICS scores with leptin levels supports this further; i.e., higher levels of chronic stress are associated with higher leptin levels. Our findings support this, showing a strong trend towards significance when examining the influence of childhood trauma on leptin levels (more trauma leading to higher levels of leptin). Further support for this assumption is offered by the significantly mediating effect of leptin on chronic stress and childhood trauma.

Taken together, the neurohumoral profile observed in patients with MSD can be a strong indication of underlying HPA-axis dysfunction as part of a dysregulated stress response. Supporting data is provided by significant correlations between TICS scores and cortisol as well as leptin levels in addition to the significant influence of the degree of childhood trauma on these variables. The complex interplay between leptin and cortisol prohibits a conclusive statement as to the direction of the observed effects. It seems likely, however, that the observed phenotype of patients with MSD is strongly associated with the findings of HPA-axis dysfunction as expressed by hypocortisolism and hyperleptinemia through chronic stress, altered inflammatory response state, and reduced feelings of satiety. Through chronic stress, the allostatic overload of these systems leads to a static state of chronic overactivation as opposed to their natural dynamic adaptive response. The high prevalence of pain in patients with MSD due to higher leptin levels, as discussed above, is also plausible and could be one of the underlying mechanisms of stress-induced hyperalgesia often observed in patients with FSS and FMS [12].

In a multisystem approach, it could be hypothesized that alterations of the endocannabinoid system (ECS) which regulates both the stress response [47] and metabolic functions of the organism [48] could contribute to the dysregulation of the HPA axis and the development of hypothalamic leptin and insulin resistance [49]. These ECS-related dysfunctions resulting from feed-forward mechanisms of prolonged stress and excessive food intake could very well explain the stronger relationship between leptin concentration and BMI in patients with MSD. Further studies are needed to elucidate the role of chronic stress and the ECS in MSD.

The limitations of our study include the cross-sectional nature of its design precluding causal inference as to the cause and effect of the observed changes and lack of 24-hour cortisol profiles. The difference in medication use between patients and controls could also be involved in hormonal alterations but is inherent to the use of a healthy control group. On the other hand, in the patient group, no significant differences on the hormone levels were found between the subgroups of no medication, conventional analgesics, and centrally acting compounds. Additionally, the small number of male participants limits generalizability to male patients.

In conclusion, our study underlines common alterations in hormone levels in female patients with MSD that are also seen in several other distinct chronic pain states suggesting common mechanisms. Our findings give support to the central role of a dysregulated response to chronic stress, ACE, and metabolic balance as well as a state of hypocortisolism and HPA-axis dysfunction in patients diagnosed with MSD. Future studies should prospectively investigate the complex interplay of leptin, IGF-1, sex hormones, and cortisol further. If possible, such studies should include the measurement of 24 h variation of hormone levels and their response to experimental stressors.

## Figures and Tables

**Table 1:** Group characteristics of study participants. Values are whole numbers or represented as mean ± SD (standard deviation).

	Male				Female			
	Controls		Patients		Controls		Patients	
	Mean	SD ±	Mean	SD ±	Mean	SD ±	Mean	SD ±
Age (years)	46.06	14.44	51.67	9.38	53.32	8.72	55.14	10.11
Body mass index	25.02	3.38	26.63	3.75	24.20	3.96	27.02	5.26
SF-36 physical sum scale	55.11	3.57	30.09	7.75	53.86	5.95	28.62	7.83
SF-36 psychological sum scale	50.40	8.05	33.97	15.63	53.32	6.57	39.01	12.05
VAS 0 bis 10	1.17	2.27	7.12	1.50	1.82	2.62	7.00	1.55

**Table 2:** Results of plasma hormone levels in patients and controls. Values are represented as mean ± SD (standard deviation). There were significant differences between leptin, FSH, IGF-1, cortisol, and BMI between female patients and controls but not in male participants as indicated.

	Male participants							Male participants						
	Control group			Patients				Control group			Patients			
	Mean	Standard deviation ±	*n*	Mean	Standard deviation ±	*n*	*p*=	Mean	Standard deviation ±	*n*	Mean	Standard deviation ±	*n*	*p*=
Cortisol (*μ*g/dl)	15.10	4.31	18	14.18	5.57	13	0.61	16.55	4.91	130	14.76	4.55	137	0.002
LH (U/l)	3.98	2.08	18	9.33	21.43	13	0.298	22.40	15.85	130	25.20	15.26	137	0.142
FSH (mU/ml)	4.61	2.92	18	17.88	39.19	13	0.16	44.08	36.67	130	53.16	37.38	137	0.046
IGF-1 (ng/ml)	158.67	57.76	18	135.77	55.73	13	0.278	144.61	44.18	130	129.64	64.81	137	0.029
DHEA-S (ng/ml)	2.078, 28	1.227, 16	18	1.731, 83	1.391, 21	12	0.478	988.97	555.95	130	897.01	665.89	137	0.224
Leptin (ng/ml)	7.59	5.11	12	13.76	22.50	12	0.365	15.76	12.13	129	25.24	18.08	91	<0.001
Body mass index	25.02	3.38	16	26.63	3.75	12	0.244	24.20	3.96	125	27.02	5.26	123	<0.001

## Data Availability

The data supporting these findings are available from the corresponding author, JA, upon reasonable request.

## Supplementary Materials

Supplementary Materials Table S1: overview of medication use of patients and controls according to gender. Figure S1: BMI frequency distribution of female patients and controls.
