# Supplementary material for: Neurohumoral Profiles and Childhood Adversity of Patients with Multisomatoform Disorder and Pain as the Leading Bodily Symptom
Source: Dis Markers. 2022 Feb 22;2022:7958375. doi: 10.1155/2022/7958375 (PMC8888045; doi:10.1155/2022/7958375)
Supplement: Supplementary Materials — Table S1: overview of medication use of patients and controls according to gender. Figure S1: BMI frequency distribution of female patients and controls. [file 7958375.f1.docx]

Supplementary description:

Table S1: Overview of medication use of patients and controls according to gender.

Figure S1: BMI frequency distribution of female patients and controls.
